# Serum FoxO1 and SIRT2 concentrations in healthy pregnant women and complicated by preeclampsia

**DOI:** 10.1007/s11845-024-03865-5

**Published:** 2025-01-14

**Authors:** Asuman Akkaya Fırat, Ebru Alıcı Davutoğlu, Aysegül Özel, Serap Fırtına Tuncer, Nevin Yılmaz, Rıza Madazlı

**Affiliations:** 1 Department of Medical Biochemistry, Istanbul University Cerrahpasa Faculty of Medicine, Istanbul, Turkey; 2 Department of Obstetrics and Gynecology, Perinatology Clinic, Istanbul University Cerrahpasa Faculty of Medicine, Istanbul, Turkey; 3 Department of Obstetrics and Gynecology, Antalya Training and Research Hospital, Antalya, Turkey

**Keywords:** Apoptosis, FoxO1, Preeclampsia, Placental senescence, SIRT2, Trophoblast invasion

## Abstract

**Background:**

Sirtuins and FoxO1 are reported to be important in the pathophysiology of preeclampsia. This study aimed to investigate whether serum FoxO1 and SIRT2 concentrations differ between preeclampsia and normal pregnancy and also to compare these markers in early- and late-onset preeclampsia.

**Methods:**

This cross-sectional study was conducted on 27 women with early-onset preeclampsia, 27 women with late-onset preeclampsia, and 26 healthy normotensive pregnant controls. Maternal serum levels of FoxO1 and SIRT2 were measured with the use of an enzyme-linked immunosorbent assay kit.

**Results:**

The mean maternal serum FoxO1 levels were significantly lower both in early-onset (9.1 ± 3.8 vs. 29.1 ± 3.2, *p* < 0.001) and late-onset preeclampsia (2.6 ± 1.6 vs. 29.1 ± 3.2, *p* < 0.001) than the normotensive pregnancies. The mean maternal serum FoxO1 level of late-onset preeclampsia was significantly lower than the early-onset preeclampsia group (2.6 ± 1.6 vs. 9.1 ± 3.8, *p* < 0.001). The mean maternal serum SIRT2 levels were significantly lower both in early-onset (4.5 ± 2.1 vs. 6.3 ± 0.9, *p* < 0.001) and late-onset preeclampsia (2.1 ± 0.6 vs. 6.3 ± 0.9, *p* < 0.001) than the healthy pregnancies.

**Conclusions:**

FoxO1 and SIRT2 may be biomarkers for early detection of preeclampsia and potential therapeutic targets in the pathophysiology of preeclampsia**.**

## Introduction

Pre-eclampsia (PE) is a leading cause of maternal and neonatal mortality, occurring in approximately 2–8% of all pregnancies [1]. The clinical manifestations of preeclampsia range from arterial hypertension, proteinuria, and impaired liver function to pulmonary edema, renal failure, and cerebral edema, beginning at 20 weeks gestation [2].The pathogenesis of PE has not yet been definitively established, but it is increasingly recognized that it is multifactorial and that vascular endothelial cell dysfunction at the interface of the fetal and maternal circulation contributes to the development of physiopathologic mechanisms [3]. In clinical diagnosis, according to the time of onset of the disease about gestational age, cases that occur before 34 weeks of gestation are classified as early-onset (EO-PE) and cases that occur at 34 weeks of gestation or later are classified as late-onset preeclampsia (LO-PE) [4]. EO-PE in particular occurs as a result of incomplete and incomplete trophoblast invasion and placentation and carries an increased risk of severe neonatal and maternal complications and fetal death [5, 6]. LO-PE has mild clinical symptoms, rather than inadequate placentation, and is usually caused by predisposing maternal factors and mainly by the mother’s overreaction to pregnancy [2, 6].

FoxO1 proteins are a subclass of the large family of Forkhead transcriptional regulators that control the regulation of many genes involved in fundamental cellular processes, including cell cycle regulation, cell death, modulation of inflammation, metabolism, protection from oxidative stress, and survival [7]. FoxO1 is expressed in the syncytiotrophoblasts (STBs) of chorionic villi and its acetylated (ac-FoxO1) and phosphorylated (p-FoxO1) forms are expressed in both STBs and cytotrophoblasts (CTBs) [7]. Aberrant trophoblast cell turnover occurs in PE, leading to increased apoptosis in placental trophoblasts [8]. FoxO1 is critical for trophoblast function and apoptosis and abnormal FoxO1 expression may contribute in part to the abnormal trophoblast differentiation and apoptosis in PE [9, 10].

The silent information regulator 2 (SIRT2) is a member of the family of Sirtuins, which are NAD^+^-dependent deacetylase regulators of several biological processes such as DNA recombination, gene silencing, DNA repair, chromosome stability, long survival, and necroptosis [11]. SIRT2 is greatly expressed in decidual cells, CTBs, STBs, placental endothelium, and amniotic epithelial cells [12]. Sirtuins play key roles in the enhancement of trophoblast survival, differentiation, and invasion during pregnancy [13]. Sirtuins can improve the pregnancy outcome by reducing placental inflammation and oxidative stress and enhancing cell survival, placental angiogenesis, trophoblast differentiation, and invasion [12]. Alterations in Sirtuin levels may be a pivotal intermediary step in the pathogenesis of several pregnancy disorders such as recurrent spontaneous abortion, PE, and fetal growth restriction (FGR) [12, 14]. PE is related to inappropriate maternal inflammatory response, oxidative stress, and vascular endothelial cell dysfunction during pregnancy.

Both FoxO1 and Sirtuins are present in the human placenta, particularly in trophoblast cells, and appear to be important for regular placenta formation, development, and adequate trophoblastic invasion [15–17]. FoxO1 and SIRT2 are known to play a role in protective mechanisms against inflammation, oxidative stress, cellular senescence, and apoptosis in various pathological conditions [18]. This study aimed to analyze the involvement of FoxO1 and SIRT2 in the PE process. We tried to evaluate the relationship between maternal serum levels of these biologically active compounds in EO-PE and LO-PE and determine their possible effects on the clinical course.

### Ethical clearance

This case–control study was initiated and conducted in the Department of Obstetrics and Gynecology, Cerrahpaşa Medical Faculty. The Ethics Committee of our university approved the study protocol by the Declaration of Helsinki (approval number: 284319). All participants were given detailed instructions and signed informed consent forms before enrollment.

## Methodology

We enrolled 26 women with healthy pregnancies, 27 pregnant women with EO-PE, and 27 pregnant women with LO-PE. We performed our study with 80 patients with a 90% confidence interval according to power analysis. Exclusion criteria were multiple pregnancies, premature rupture of membranes, chorioamnionitis, or medical complications such as autoimmune disorders, diabetes mellitus, smoking, chronic hypertension, polyhydramnios, inflammation, and previous renal diseases. No pathologic process was detected in cases with healthy pregnancies and all of them gave birth. To diagnose PE, it was defined as new-onset hypertension (≥ 140/90 mm Hg) and proteinuria (≥ 2 on the dipstick or ≥ 300 mg in 24 h in a 24-h urine sample) observed in at least two separate measurements 6 h or more apart. The amount of excreted protein was analyzed in 24-h urine samples. Gestational age determination was based on ultrasound measurement of fore-aft length in the first trimester. FGR was diagnosed when the fetal abdominal circumference was found to be less than 2 standard deviations from the mean for gestational age, and this finding was detected by sequential ultrasonographic measurement of fetal growth.

### Materials

Before starting treatment, venous blood samples were taken from EO-PE, LO-PE, and healthy pregnant women at the time of admission to the hospital. None of the patients were in labor at the time of sampling. For the analysis of biomarkers (FoxO1 and Sirtuin2), serum was obtained by centrifuging clotted samples from venous blood samples within 30 min. Separated serum samples were stored in several small aliquots at 80 °C until analysis. The enzyme-linked immunosorbent assay (ELISA) procedure was used to determine the levels of markers in sera (in duplicate). ELISA tests (Sunred Biotechnology Co. Aviscera Bioscience Inc., Shanghai, China. Catalog No: 201–12–0629) were used for FoxO1 and (MyBioSource San Diego, California, ABD Catalog No: MBS2530369) for Sirtuin2. In our study, all determinations were done according to the manufacturer’s instructions. At the final step of the procedure, the absorbances were measured in a microplate reader at 450 nm. By plotting a standard curve, from known concentrations versus measured absorbances, FoxO1 levels were expressed as ng/mL and ng/mL for Sirtuin2. The measuring ranges, sensitivity, intra-assay precision, and inter-assay precision of the kits were 0.15–40 ng/mL, < 10%, < 12% for FoxO1, and 0.78–50 ng/mL, 0.19 ng/mL < 10%, < 12 for Sirtuin2.

### Statistical analysis

All statistical analyses were calculated using Statistical Package for the Social Science (SPSS) software version 21 (Chicago, IL, USA). The Kolmogorov–Smirnov test was applied to assess whether the distribution of the variables of the cases was normal. All values are expressed as mean ± standard deviation. Descriptive statistics for the characteristics of the subjects were calculated for both continuous and categorical variables. Comparisons between all groups were calculated using the Kruskal–Wallis test or the one-way ANOVA test. The significance of differences between variables in subgroups (EO-PE, LO-PE, and controls) was analyzed by ANOVA/Bonferroni test. The comparison value was considered statistically significant when the *p* value was < 0.05.

## Results

The clinical and demographic characteristics and perinatal outcomes of the study groups are given in Tables 1 and 2. There was no significant difference between the study groups for mean maternal age, incidence of nulliparity, and number of platelets (*p* > 0.05). There was a significant difference between all three groups for gestational age at blood sampling, diastolic blood pressure, mean uterine artery PI, and birth weight (*p* < 0.01). Body mass index and systolic blood pressure values were significantly higher in the preeclamptic groups than in the control group (*p* < 0.01). Gestational age at delivery was significantly lower in the EO-PE group than in the LO-PE and control groups (*p* < 0.01). Incidences of FGR and admission to NICU were significantly higher in the EO-PE group than in the LO-PE group (*p* < 0.01). There was no stillbirth or early neonatal death in LO-PE and control pregnancies. Stillbirth and early neonatal death rates were significantly higher in EO-PE than in LO-PE and control pregnancies (*p* < 0.001). The 2 stillbirths occurred at 25 and 26 weeks of gestation. The early neonatal deaths were delivered at 26 and 27 weeks of gestation death was due to complications of perinatal asphyxia and prematurity.

**Table 1:** Maternal characteristics of the study groups

	Early-onsent preeclampsia	Late-onsent preeclampsia	Control	*p*^1^	*p*^2^	*p*^3^
*N*	27	27	26			
Age (years)	31.6 ± 5.5	29.4 ± 5.2	28.8 ± 5.3	0.183	1.0	0.445
Nulliparous	7 (26.0)	12 (44.4)	11 (42.3)	0.441	0.986	0.346
Gestational age at blood sampling (weeks)	28.8 ± 3.7	36.1 ± 1.7	38.3 ± 1.4	< 0.001^*^	0.005^*^	< 0.001
Body mass index at blood sampling (kg/m^2^)	31.2 ± 4.8	31.9 ± 4.6	27.9 ± 1.6	0.013^*^	0.002^*^	1.0
SBP (mmHg)	156.8 ± 12.2	155.1 ± 12.9	114.2 ± 4.6	< 0.001^*^	< 0.001^*^	1.0
DBP (mmHg)	104.1 ± 10.4	97.6 ± 6.9	69.6 ± 5.3	< 0.001^*^	< 0.001^*^	0.01^*^
Mean UtA PI	1.64 ± 0.34	1.03 ± 0.23	0.75 ± 0.21	< 0.001^*^	< 0.001^*^	< 0.001^*^
Platelets (× 10^9^/I)	215.8 ± 62.4	195.4 ± 83.9	226.5 ± 61.4	1.0	0.349	0.865

Values are given as mean ± SD or number (%)

*SBP* systolic blood pressure, *DBP* diastolic blood pressure, *PI* pulsatility index, *UtA* uterine artery, *FGR* fetal growth restriction, *NICU* neonatal intensive care unit

*p*^1^: *p* values of the comparison of control and EO-PE groups; *p*^2^: *p* values of the comparison of control and LO-PE groups; *p*^3^: p values of the comparison of the EO-PE and LO-PE groups (Kruskal–Wallis, one-way ANOVA test)

^*^p value < 0.05 is considered statistically significant

**Table 2:** Fetal characteristics of the study groups

Gestational age at delivery (weeks)	30.8 ± 3.3	37.4 ± 1.6	38.4 ± 1.5	< 0.001*	0.277	< 0.001*
Birth weight (g)	1386 ± 756	2825 ± 670	3334 ± 467	< 0.001*	0.016*	< 0.001*
FGR	18 (66.6)	9 (33.3)	-	-	-	0.006**
Admission to NICU	21 (77.8)	5 (18.5)	-	-	-	< 0.001**
Stillbirth	2 (7.4)	-	-	< 0.001*	-	< 0.001*
Early neonatal death	2 (7.4)	-	-	< 0.001*	-	< 0.001*
Perinatal mortality	4 (14.8)	-	-	< 0.001*	-	< 0.001*

Values are given as mean ± SD or number (%)

*SBP* systolic blood pressure, *DBP* diastolic blood pressure, *PI* pulsatility index, *UtA* uterine artery, *FGR* fetal growth restriction, *NICU* neonatal intensive care unit

*p*^1^: *p* values of the comparison of the control and EO-PE groups; *p*^2^: *p* values of the comparison of the control and LO-PE groups; *p*^3^: *p* values of the comparison of the EO-PE and LO-PE groups (Kruskal–Wallis, one-way ANOVA test)

^*^p value < 0.05 is considered statistically significant

**Fig. 1:**
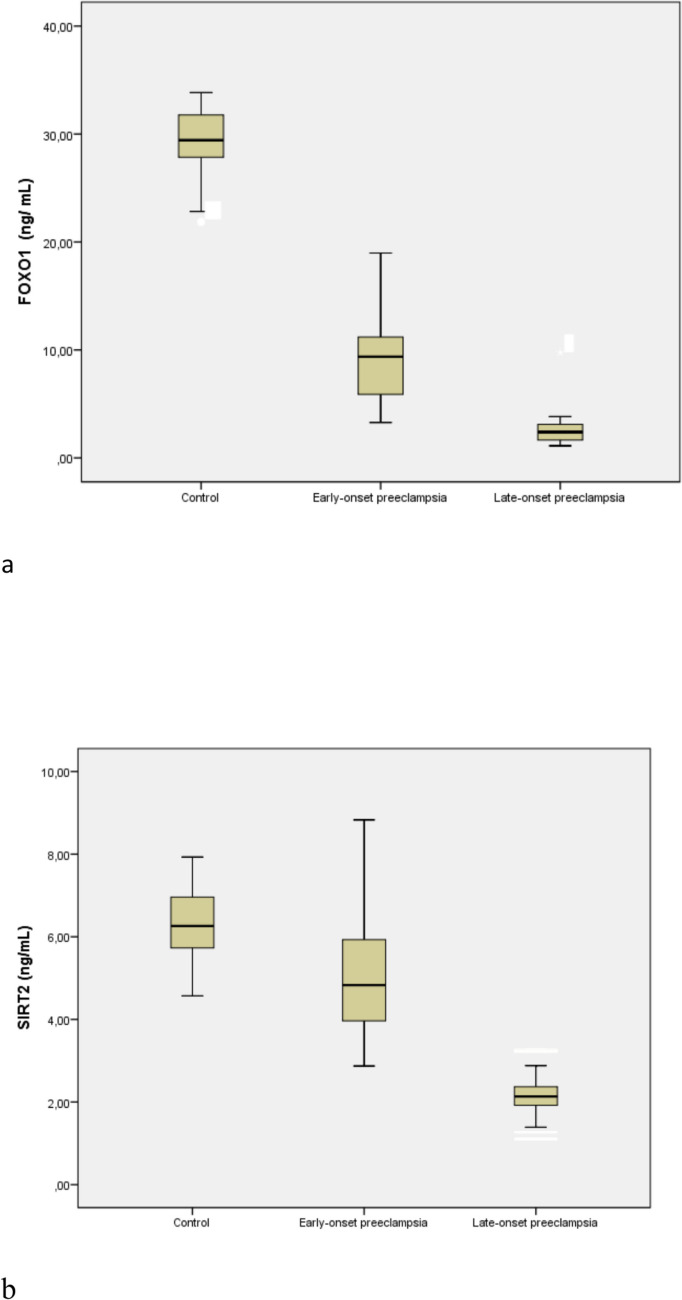
Boxplot analysis of maternal serum markers. Patients with early-onset preeclampsia, late-onset preeclampsia, and a control group were compared for FOXO1 (**a**) and SIRT2 (**b**)

The mean maternal serum FoxO1 and SIRT2 levels of the control, EO-PE, and LO-PE groups are demonstrated in Table 3. Box plot graphics of maternal serum FoxO1 and SIRT2 levels of the study groups are also illustrated in Fig. 1. The mean maternal serum FoxO1 levels were significantly lower both in EO-PE (9.1 ± 3.8 vs. 29.1 ± 3.2, *p* < 0.001) and LO-PE (2.6 ± 1.6 vs. 29.1 ± 3.2, *p* < 0.001) than the normotensive pregnancies. The mean maternal serum FoxO1 level of LO-PE was significantly lower than the EO-PE group (2.6 ± 1.6 vs. 9.1 ± 3.8, *p* < 0.001). The mean maternal serum SIRT2 levels were significantly lower both in EO-PE (4.5 ± 2.1 vs. 6.3 ± 0.9, *p* < 0.001) and LO-PE (2.1 ± 0.6 vs. 6.3 ± 0.9, *p* < 0.001) than the normotensive pregnancies. The mean maternal serum SIRT2 level of LO-PE was significantly lower than the EO-PE group (2.1 ± 0.6 vs. 4.5 ± 2, *p* < 0.001). There was also a good correlation between maternal serum FoxO1 and SIRT2 levels (*r* = 0.716, *p* < 0.001).

**Table 3:** Maternal serum FoxO1 and SIRT2 levels of control, early-onset- and late-onset -preeclampsia groups

	Early-onset preeclampsia	Late-onset preeclampsia	Control	*p*^1^	*p*^2^	*p*^3^
*N*	27	27	26			
FoxO1 (ng/mL)	9.1 ± 3.8	2.6 ± 1.6	29.1 ± 3.2	0.000	0.000	0.000
Sirtuin2 (ng/mL)	4.5 ± 2.1	2.1 ± 0.6	6.3 ± 0.9	0.000	0.000	0.000

Values are given as mean ± SD

*p*^1^: *p* values of the comparison of the control and EO-PE groups; *p*^2^: *p* values of the comparison of the control and LO-PE groups; *p*^3^: *p* values of the comparison of the EO-PE and LO-PE groups (Kruskal–Wallis, Bonferroni test)

*p*-value < 0.05 is considered statistically significant

**Fig. 2:**
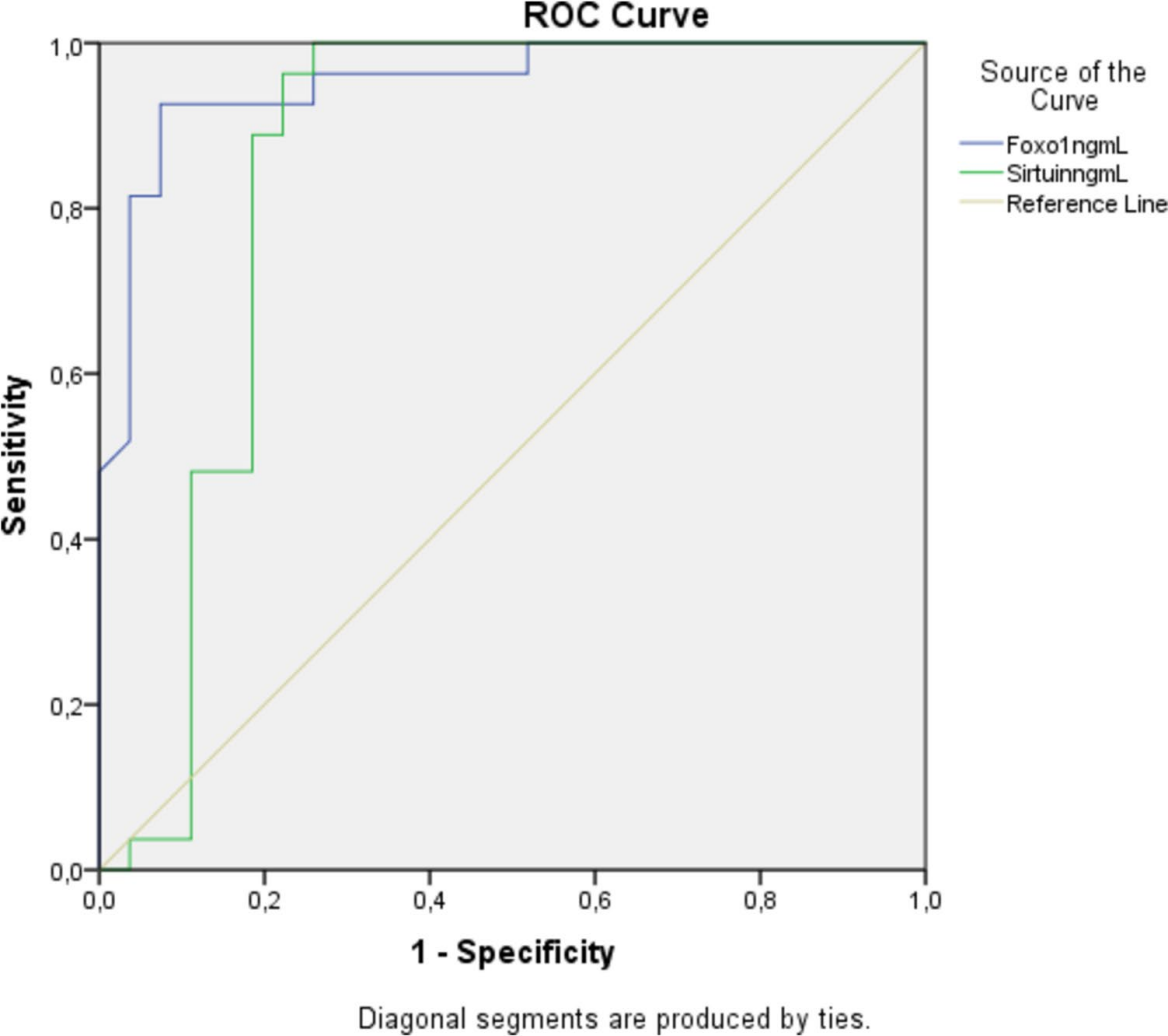
The ROC curve analysis of maternal serum FoxO1 and SIRT2 concentration for predicting preeclampsia

Receiver operating characteristic (ROC) curves were also evaluated. The threshold value for maternal serum FoxO1 in predicting preeclampsia < 3.64 ng/mL, and the area under the curve (AUC) was 0.951 (95% confidence interval (CI) 0.82–0.93, 0.875, *p* > 0.001 with 78.8% sensitivity and 76.5% specificity). The threshold value for maternal serum Sirtuin2 in preeclampsia < 2.76 pg/mL, and the area under the curve (AUC) was 0.848 (95% confidence interval (CI) 0.85–0.97, 0.91, *p* > 0.001 with 80.2% sensitivity and 76.5% specificity) (Fig. 2). The ROC curve analysis of maternal serum SIRT2 and FoxO1 concentration for predicting preeclampsia.

## Discussion

To our knowledge, this study is the first to compare maternal serum FoxO1 and SIRT2 levels in women with EO-PE, LO-PE, and normotensive pregnancies. Our results showed that serum FoxO1 and SIRT2 levels were significantly lower in the PE group compared to the control group. Maternal serum FoxO1 and SIRT2 levels were also significantly lower in pregnancies with LO-PE than EO-PE.

Healthy placentation relies on a precise balance between trophoblast proliferation and apoptosis [19]. Altered apoptosis has been identified in placentas from PE pregnancies and FGR [20]. FOXO family regulates gene expression involved in growth, autophagy, differentiation, cell proliferation, and motility through interaction with the promoter site of its target genes [21]. Chen et al. reported that FoxO1 is an important switching agent for apoptosis and autophagy in endometrial epithelial cells of PE cases due to oxidative stress [22]. Alzubaidi et al. demonstrated that there was significant upregulation of FoxO1, FoxO3a, FoxO4, and FoxO6 gene expression in PE women compared to the healthy pregnant group [21]. In the same study, LO-PE patients showed increased mRNA expression of FoxO1, FoxO3a, FoxO4, and FoxO6 compared to EOPE patients [21]. They speculated that increased expression of FoxO genes may be related to increased apoptosis and autophagy seen in PE. Sheridon et al. in an immunohistochemical study performed on placentas with mild preeclampsia found that the ratio of FoxO1-positive and FoxO1-negative nuclei in STB cell nuclei was significantly lower than the control group [10]. However, there was no significant difference between the rates of STB cells containing FoxO1-positive/negative nuclei of the severe preeclampsia group compared to the control group [10]. Zhang et al. demonstrated that FoxO1-related proteins were decreased in the placenta of PE patients and were closely related to the expression of oxidative stress, inflammatory response, endothelial damage factors, and apoptotic molecules [9]. The results of FoxO1gene activity and levels of FoxO1-related proteins in PE are conflicting and inconclusive. In a comprehensive bioinformatic gene analysis of placental tissue from early-onset and late-onset pre-eclampsia, Liu et al. highlighted that inadequate function of FoxO1 and blockade of the AMPK pathway lead to more severe consequences for the fetus, especially in early-onset preeclampsia [23]. However, we may speculate that reduced maternal serum levels of FoxO1 demonstrated in our study could be associated with abnormal trophoblast differentiation and apoptosis in PE.

Sirtuins are a conserved family of proteins that are involved in the regulation of several physiological conditions such as apoptosis, inflammatory response, stress, and aging [12]. It has been shown that deficiency in each member of the Sirtuin family results in severe developmental defects and irregularities both in animals and humans [11, 12]. McBurney showed that mice carrying two null alleles of Sirtuin were smaller than normal at birth, and most died during the early postnatal period [24].

Most of the studies regarding Sirtuins and PE involve SIRT1. SIRT1 is lower in PE placentas and serum samples and is mainly expressed in the CTBs and STBs [25, 26]. SIRT1 might affect placental development and trophoblast invasion through autophagy and senescence in PE, and SIRT1 protects vascular endothelial cells from oxidative stress, inflammatory response, autophagy, and senescence [25]. SIRT2 has been less studied than other Sirtuin family members in PE. SIRT2, like SIRT1, is also found in the cytoplasm, mitochondria, and nucleus of the decidual cells, CTBs, STBs, placental endothelium, and amniotic epithelial cells [12]. SIRT2 regulates important physiological functions for the cell and plays a role in many cancers and neurodegenerative diseases [12]. Hannan et al. showed a significant reduction in SIRT2 protein expression in both PE and FGR placentas relative to gestation-matched controls [19]. In preeclamptic placentas, this reduction was illustrated to be concomitant with elevated mRNA levels of receptor-interacting serine/threonine-protein kinase 1 (RIPK1), an enzyme involved in the induction of necroptosis and they concluded that necroptosis may contribute to placental pathophysiology that underlies PE [19]. By this study, we have also observed decreased serum SIRT2 levels in PE women.

In the present study, maternal serum FoxO1 and SIRT2 levels were significantly lower in women with LO-PE than EO-PE. Sirtuins, also known as longevity genes, have functions linked to cellular aging and survival [25, 27]. Maternal serum Sirtuin levels may decrease as a result of placental senescence. We speculate that the cause of the more significant reduction of both maternal SIRT 2 and FoxO 1 levels observed in LO-PE than EO-PE may in part be due to the placental senescence of more advanced placentas in LO-PE. Also, these striking differences in maternal serum FoxO1 and SIRT2 levels between EO-PE and LO-PE suggest that these two conditions are not only clinically diverse but also may have different pathophysiological mechanisms [28, 29]. While the pathophysiology of early-onset preeclampsia is inappropriate placental formation and development, the possible cause of late-onset preeclampsia may be premature placental aging; therefore, regardless of gestational week, FoxO1 and SIRT 2 levels may be decreased more in the late-onset group compared to both the early-onset preeclampsia group and the healthy group.

Our study group could be completed with the number of cases required for a 90% confidence interval. This is below the sample size required for a 95% confidence interval and constitutes the main limitation of our study. There is a need for studies with larger case groups that are more reflective of the whole population.

## Conclusion

Reference intervals for FoxO1 and SIRT2 for all gestational weeks are not yet available in the literature. Maternal serum FoxO1 and SIRT2 levels are decreased in PE pregnancies, with a more significant decrease in LO-PE than EO-PE. The difference in FoxO1 and SIRT 2 serum levels in early- and late-onset preeclampsia does not seem to be related to the gestational week but probably to the different pathophysiologic basis of these two conditions. Early-onset preeclampsia seems to be dominated by inadequate trophoblastic invasion, whereas late-onset preeclampsia seems to be dominated by biomarkers of premature placental aging. These molecules may be important as diagnostic markers or therapeutic targets in the pathophysiology of EO-PE and LO-PE. Further research is needed to better understand the importance and effects of FoxO1 and SIRT2 on PE.

## Data Availability

All data generated or analyzed during this study are included in this article. Inquiries can be directed to the corresponding author at asumanfirat44@gmail.com.
